# Applying multi-omics data to study the genetic background of bovine respiratory disease infection in feedlot crossbred cattle

**DOI:** 10.3389/fgene.2022.1046192

**Published:** 2022-12-12

**Authors:** Jiyuan Li, Robert Mukiibi, Janelle Jiminez, Zhiquan Wang, Everestus C. Akanno, Edouard Timsit, Graham S. Plastow

**Affiliations:** ^1^ Livestock Gentec, Department of Agriculture, Food & Nutritional Science, University of Alberta, Edmonton, AB, Canada; ^2^ The Roslin Institute and Royal (Dick) School of Veterinary Studies, University of Edinburgh, Edinburgh, Scotland, United Kingdom; ^3^ Faculty of Veterinary Medicine, University of Calgary, Calgary, AB, Canada

**Keywords:** disease susceptibility, GWAS, differential expressed gene, eQTL, causal SNP, metabolomics

## Abstract

Bovine respiratory disease (BRD) is the most common and costly infectious disease affecting the wellbeing and productivity of beef cattle in North America. BRD is a complex disease whose development is dependent on environmental factors and host genetics. Due to the polymicrobial nature of BRD, our understanding of the genetic and molecular mechanisms underlying the disease is still limited. This knowledge would augment the development of better genetic/genomic selection strategies and more accurate diagnostic tools to reduce BRD prevalence. Therefore, this study aimed to utilize multi-omics data (genomics, transcriptomics, and metabolomics) analyses to study the genetic and molecular mechanisms of BRD infection. Blood samples of 143 cattle (80 BRD; 63 non-BRD animals) were collected for genotyping, RNA sequencing, and metabolite profiling. Firstly, a genome-wide association study (GWAS) was performed for BRD susceptibility using 207,038 SNPs. Two SNPs (Chr5:25858264 and BovineHD1800016801) were identified as associated (*p*-value <1 × 10^−5^) with BRD susceptibility. Secondly, differential gene expression between BRD and non-BRD animals was studied. At the significance threshold used (log_2_FC>2, logCPM>2, and FDR<0.01), 101 differentially expressed (DE) genes were identified. These DE genes significantly (*p*-value <0.05) enriched several immune responses related functions such as inflammatory response. Additionally, we performed expression quantitative trait loci (eQTL) analysis and identified 420 cis-eQTLs and 144 trans-eQTLs significantly (FDR <0.05) associated with the expression of DE genes. Interestingly, eQTL results indicated the most significant SNP (Chr5:25858264) identified *via* GWAS was a cis-eQTL for DE gene *GPR84.* This analysis also demonstrated that an important SNP (rs209419196) located in the promoter region of the DE gene *BPI* significantly influenced the expression of this gene. Finally, the abundance of 31 metabolites was significantly (FDR <0.05) different between BRD and non-BRD animals, and 17 of them showed correlations with multiple DE genes, which shed light on the interactions between immune response and metabolism. This study identified associations between genome, transcriptome, metabolome, and BRD phenotype of feedlot crossbred cattle. The findings may be useful for the development of genomic selection strategies for BRD susceptibility, and for the development of new diagnostic and therapeutic tools.

## Introduction

Bovine respiratory disease (BRD) is a worldwide infectious disease that causes a significant economic loss in cattle production due to mortality, increased expenses associated with prevention, treatment and labour, impaired growth of animals, and reduced carcass value (Griffin, 1997; Smith, 2000; Irsik et al., 2006; Schneider et al., 2010). In North America, BRD has been identified as the most expensive infectious disease in the beef cattle industry (Taylor et al., 2010), causing high morbidity rates that can reach up to 80%, and moderate to high mortality in some feedlots (Smith, 1998; Baptista et al., 2017). Newly-received cattle in the feedlot are highly susceptible to BRD due to compromised immunity from a number of stressors including weaning, long-distance transportation, and co-mingling of cattle from different sources especially in auction markets (Taylor et al., 2010). These stressors expose the animals to multiple BRD pathogens and provide a conducive environment for the emergence of opportunistic viral and bacterial infections of the respiratory tract (Griffin et al., 2010; Taylor et al., 2010; Kirchhoff et al., 2014). In feedlots, the most commonly used disease control approach is treating animals with a wide range of antibiotics before or on entry into the feedlots (Ives and Richeson, 2015), however this may lead to the development of antimicrobial resistance which is a major concern for both human and animal health (Klima et al., 2014; Stanford et al., 2020). Additionally, early diagnosis and appropriate treatment of infected animals could increase recovery from infections and potentially reduce negative impacts of the disease on animal performance and productivity. However, most clinical signs of BRD are subjective, difficult to standardize, and non-specific for BRD, which makes the diagnosis of BRD difficult (Buczinski and Pardon, 2020).

Heritability estimates of BRD range from 0.07 to 0.29 (Snowder et al., 2005; Schneider et al., 2010; Neibergs et al., 2014a; 2014b). This suggests there is the potential to breed BRD resistant animals, which would lead to a sustainable reduction in BRD incidence and potentially antimicrobial resistance (Neibergs et al., 2014b; Hoff et al., 2019). Several SNPs and quantitative trait loci (QTLs) have been reported as significantly associated with BRD through genome-wide association studies (GWAS) (Neibergs et al., 2014b; Hoff et al., 2019), however, investigations into the genetic background of resistance or susceptibility to BRD in beef cattle populations is an ongoing endeavour. In addition, RNA sequencing (RNA-Seq) offers high resolution profiling of transcriptomes of individual animals in a given sample/tissue, hence allowing the discovery of transcriptome-wide expression differences between animals with contrasting phenotypes of interest (e.g., BRD and non-BRD) (Costa-Silva et al., 2017; Hrdlickova et al., 2017). Such differential gene expression between BRD and non-BRD animals could help reveal differences in the host response to BRD infection and help to identify potential biomarkers for BRD diagnosis. Several transcriptomic studies have been conducted to investigate the gene expression differences and host response to BRD infection (Tizioto et al., 2015; Scott et al., 2020; Sun et al., 2020; Jiminez et al., 2021). These studies have shown that host animals have the ability to respond to BRD pathogen infection and the damage caused by these pathogens by altering the expression of certain key immune genes (e.g., *BPI*, *GPRA84, S100A8*, *S100A9*, and *IL3RA*). These studies further suggest that gene expression alterations may vary with different viral and bacterial pathogen infections as well as the stage of disease development. However, these analyses have mainly focused on the correlation between the transcriptome and BRD infection status, with no consideration of the potential relationship between different omics layers. Changes in gene expression are not only associated with the disease or trait of interest, but are also affected by genomic regulation (i.e., expression QTL, eQTL) (Cookson et al., 2009). Thus, integration of GWAS, differential gene expression analysis and eQTL analysis could aid in interpreting the results of GWAS and identifying functional or causal SNPs. Furthermore, metabolites are small molecules involved in metabolic activities, which could provide additional insights into the host response to disease, and could also be used as biomarkers to indicate the presence or absence of the disease (Xia et al., 2013; Blakebrough-Hall et al., 2020). Identifying metabolites associated with BRD, and studying their correlations with DE genes, may lead to a better understanding of the biological basis for disease response and could help to identify more biomarkers involved in disease pathogenesis. For BRD, however, this knowledge is currently largely unknown.

Therefore, the objectives of this study were to identify DNA markers associated with BRD susceptibility, the DE genes and significant metabolites associated with BRD infection in feedlot cattle. We also aimed to identify cis- and trans-eQTLs associated with DE genes and correlations between DE genes and significant metabolites in this study. This multi-omics study may shed light on the genetic and molecular architecture of BRD in crossbred feedlot cattle and provide information on biomarker identification in the general beef cattle population in Canada.

## Materials and methods

### Animal population and phenotype collection

A total of 143 crossbred or multi-breed beef cattle were used in this study. Animals were conventionally raised cattle that included heifers (*n* = 87) and steers (*n* = 56). These animals were enrolled into the feedlot during the fall of 2015 at four commercial feedlots in Central/Southern Alberta, Canada. On-arrival processing of the animals was previously described (Jiminez et al., 2021). Briefly, animals were weighed (average body weight: 571 ± 106 Lb) and received a subcutaneous injection of a long-acting macrolide (tulathromycin, Draxxin, 2.5 mg/kg, Zoetis, Kirkland, QC, Canada) and vaccinated against multiple bacterial and viral agents including bovine herpes virus-1 (BoHV-1), bovine viral diarrhea virus (BVDV) (types I and II), bovine parainfluenza-3 (PI3V), bovine respiratory syncytial virus (BRSV), *Mannheimia haemolytica*, *Histophilus somni*, and clostridial pathogens. They were also dewormed with a pour-on ivermectin solution. While in the feedlots, animals were fed twice daily on a concentrate barley-based receiving/growing diet. This diet also contained 25 ppm of monensin (Rumensin 200, Elanco, Guelph, ON, Canada) and 35 ppm of chlortetracycline (Aureomycin 220, Zoetis). Cattle received a growth implant and a second vaccination against infectious viruses at approximately 30 days after arrival to the feedlot. Animals were monitored daily and those that showed signs and symptoms of BRD (depression, nasal or ocular discharge, cough, tachypnea, or dyspnea) within 50 days of arrival at the feedlots were clinically examined by an experienced veterinarian. The details of clinical examinations and case definition (i.e., BRD or non-BRD cattle) were previously described (Jiminez et al., 2021). Briefly, animals were retrospectively identified as BRD positive based on clinical examination and serum haptoglobin concentration. The animal was confirmed as BRD positive if the animal displayed at least one visual BRD symptom, had a rectal temperature ≥40°C, abnormal lung sounds detected at auscultation, a serum haptoglobin concentration ≥0.25 g/L, and had no prior treatment against BRD or other diseases during the feedlot period (i.e., first BRD occurrence). For each animal that was BRD positive blood samples were collected by jugular vein puncture using Tempus (Thermo Fisher Scientific, ON), EDTA and heparin tubes (BD, Mississauga, Canada). In addition, in each pen that had a BRD positive animal, blood samples were similarly collected from two healthy matched-control or non-BRD cattle (i.e., animals which had no visual signs of BRD or other diseases, a rectal temperature <40°C, no abnormal lung sounds detected at auscultation and a serum haptoglobin concentration <0.25 g/L). A large proportion of apparently healthy pen-mates had rectal temperature >40°C, abnormal lung sounds at auscultation and/or serum haptoglobin ≥0.25 g/L. However, it is expected to find a large proportion of apparently healthy cattle with either fever, abnormal lung sounds at auscultation or high serum haptoglobin as subclinical BRD is very common early in the feeding period (Timsit et al., 2011). Therefore only 63 controls were included in this study. It was also worth noting that these 63 non-BRD cattle did not become BRD positive later during the experiment (i.e., they remained healthy until sent to market). After sample collection, animals identified as BRD positive received an antibiotic treatment subcutaneously in combination with non-steroidal anti-inflammatory drugs, in accordance with feedlot treatment protocols. The blood samples collected from both BRD positive and non-BRD animals were used for DNA extraction for GWAS analysis, transcriptome profiling for gene expression analysis and metabolome abundance profiling for the metabolomics analysis.

### DNA extraction, genotyping, and genomic breeding composition estimation

DNA was extracted from 50 uL whole blood sample of each animal using the sBeadex Livestock kit (LGC, Berlin, Germany). Four animals were not genotyped because their blood samples were lost. The extracted DNA was used to genotype each animal for 100,000 SNPs using Illumina’s GGP Bovine 100 K microarray SNP chip (Illumina, San Diego, CA, United States) by Neogen (Lincoln, NE, United States). The SNPs located on the sex chromosomes were excluded from the analysis. In addition, SNPs that had minor allele frequency <5%, or missing allele rate >10% and those that failed to pass the Hardy-Weinberg equilibrium test (*p*-value <0.0001) were also excluded from the analysis. The remaining SNPs (i.e., 85,100 SNPs) were used to predict breed composition of each animal using ADMIXTURE software (v1.3.0) (Alexander and Lange, 2011). The reason for this analysis is due to the crossbred nature of the animals in our population, which should be considered in the statistical analysis. The ancestry value of *K* = 3 was used to define the source of genetic makeup because it had the smallest cross-validation error and yielded the most accurate breed composition prediction based on prior knowledge of breed composition for a subset of animals. The genomic breed composition of each animal was shown in Supplementary Figure S1.

Additional SNP markers and their genotypes were called from the RNA-Seq data of all animals using the Genome Analysis Toolkit best practices (GATK v3.8) (Van der Auwera and O’Connor, 2020). Prior to the variant calling processes, the mapped reads from two-pass STAR alignment were sorted, had read groups added, and duplicates identified using the Picard tools package (v2.20.6). A series of processing steps including splitting “N” cigar reads (i.e., splice junction reads), reassigning mapping quality score, and base quality score recalibration were performed to improve variant calling accuracy using GATK. After data preprocessing, variants were called using the HaplotypeCaller algorithm in Genomic Variant Call Format (GVCF) mode, which included two steps: (i) variants were called individually on each sample, generating one GVCF file per sample that lists genotype likelihoods and their genome annotations; (ii) variants were called from the GVCF file through a joint genotyping analysis. The joint genotyping method is more flexible and technically easier, and is recommended for variant calling in RNA-Seq experiments (Poplin et al., 2017; Brouard et al., 2019). Stringent filtering procedures were applied to variants using the GATK Variant Filtration tool and VCFtools (v0.1.14) (Danecek et al., 2011). Indels, non-biallelic SNPs and SNPs on sex chromosomes were excluded. Then SNPs with QD < 3.0, FS > 60.0, MQ < 40.0, MQRankSum < −12.5, ReadPosRankSum < −8.0, SOR > 3.0, minor allele frequency <5%, missing allele rate > 10% and severe departure from Hardy-Weinberg equilibrium (*p*-value <0.0001) were removed.

Finally, the two SNP datasets (genotype derived vs. RNAseq derived) were merged based on the position of SNPs on the chromosome using Plink (v1.90b6.7) (Chang et al., 2015). For the overlapping SNPs in the two SNP datasets, the SNPs derived from genotyping were used. A total of 207,038 SNPs were available for 138 animals (79 BRD; 59 non-BRD animals as one sample failed to yield quality genotypes from the RNA-seq data) and used in GWAS and eQTL analysis.

### Genome-wide association analysis for BRD susceptibility

Prior to performing the GWAS for BRD susceptibility, the phenotype of BRD (BRD status of the animal, as a binary trait) was first fitted into a logistic model with a fixed effect of “feedlot,” and two covariates including days on feed, and genomic breed composition from admixture breed composition analysis. This modeling was used to determine which fixed effect and covariates had significant effects on the phenotype. Next, the GWAS analysis between SNP marker genotypes (from SNP chip and RNA-seq data) and adjusted BRD status was performed using the single SNP-based mixed linear model association (mlma), as implemented in the GCTA package (v1.93.2) (Yang et al., 2011). The linear mixed model can be described as follows:

$$y_{ij}=\mu +b_{j}x_{ij}+a_{i}+e_{ij}$$

where $y_{ij}$ is the adjusted phenotypic value of the i th animal with the j th SNP (i.e., the ij th animal), $b_{j}$ is the allele substitution effect of the j th SNP, $x_{ij}$ is the j th SNP genotype of animal i coded as 0, 1, 2 for genotypes $A_{1}A_{1}$ , $A_{1}A_{2}$ , and $A_{2}A_{2}$ , respectively, $a_{i}$ is the additive polygenic effect of the ij th animal $∼\;N\left(0,G\sigma_{a}^{2}\right)$ , and $e_{ij}$ is the random residual effect of the ij th animal $∼\;N(0,\;I\sigma_{e}^{2}$ ). The genomic relationship matrix G was derived based on total filtered SNP markers (207,038 SNPs) as described by Yang et al. (2014), which is essentially the same as VanRaden’s second formulation (VanRaden, 2008). The SNP allele substitution effect was estimated, and the significance test of the SNP allele substitution effect was conducted *via* a generalized least square F-test as implemented in the GCTA package. The phenotypic variance explained by each significant SNP was calculated by $\frac{2pq\beta^{2}}{S^{2}}*100\%$ , where p and q denote the SNP allele frequency of ${\;A}_{1\;}$ and $A_{2}$ , respectively; β is the SNP allele substitution effect; $2pq\beta^{2}$ is the additive variance of the SNP, and $S^{2}$ is the phenotypic variance.

Those SNP with FDR <0.05 were identified as significant SNPs. The threshold of *p*-value <1 × 10^−5^ were considered as the suggestive line. To visually summarize the GWAS results both the quantile-quantile (Q-Q) plot and Manhattan plot were generated using the R package qqman (Turner, 2014).

### RNA isolation, cDNA library preparation and sequencing

Tempus tubes were thoroughly mixed by 20 inversions and stored on ice until transported to the laboratory and stored at −20°C. Total RNA was extracted in two batches (Batch 1, *n* = 47; Batch 2, *n* = 96). Similar procedures were performed on all samples in both batches. Initially, total RNA was isolated from blood using a Preserved Blood RNA Purification Kit (Norgen Biotek Corp, Thorold, ON, Canada), and the quality of RNA was measured using the 2200 RNA ScreenTape TapeStation System (Agilent Technologies Inc. Cedar Creek, TX, United States) producing RNA integrity numbers (RIN) ranging from 8.0 to 9.8. Thereafter, cDNA libraries were prepared for sequencing for each individual animal from the high-quality RNA using the TruSeq RNA Library Preparation kit v2 (Illumina, San Diego, CA, United States) and the NEBNext^®^ Ultra^™^ II Directional RNA Library Prep Kit for Illumina^®^ (New England Biolabs Ltd. Whitby, ON, Canada). Samples in both batches used the stranded library preparation process. Paired-end sequencing was performed using the Hiseq 4000 platform and Novaseq 6000 for batch 1 and batch 2 samples respectively to generate paired-end sequences of 100 bp read length. Sequencing of samples in the two batches was performed at the McGill University and Genome Quebec Innovation Center (Montreal, QC, Canada). Finally, the raw reads of 143 samples (80 BRD; 63 non-BRD) were obtained and used for downstream analyses.

### Sequence data processing, alignment and counting

Raw reads for each sample were assessed for quality using FastQC (v0.11.8) (Andrews, 2010). The bases with low quality score (Phred quality score <20) and 3’ adapter sequences on raw reads were removed using Trimmomatic (v0.39) (Bolger et al., 2014). These cleaned-up sequences were aligned to the *Bos taurus* reference genome (ARS-UCD1.2.98, downloaded from Ensembl genome browser) using a short read alignment software STAR (v2.7.1a) with paired-end default parameters (Dobin et al., 2013). FeatureCounts (SubRead v1.6.4) was used to count the reads that aligned to a particular annotated gene in the bovine genome (Liao et al., 2014) and these counts were consequently used for differential gene expression analysis between BRD and non-BRD animals.

### Differential gene expression analysis and functional enrichment analysis

Read counts per gene generated by FeatureCounts as described above were utilized for differential gene expression analysis between BRD and non-BRD animals using the R Bioconductor package edgeR (McCarthy et al., 2012). Firstly, lowly expressed (count per million or CPM <0.5 in at least 63 samples) genes were filtered out from the analysis. Counts of the retained genes were then normalized using the trimmed mean M values (TMM) method (Robinson and Oshlack, 2010), to account for the technical variations between samples that may have been caused by the RNA extraction, cDNA library construction, and differences in sequencing depth (Robinson and Oshlack, 2010). The normalized counts were then modeled for differential gene expression between BRD and non-BRD animals using generalized linear models (GLM) under a negative binomial distribution assumption that considered feedlot, genomic breed composition, and sequencing batch as fixed effects. To test for significance of differential expression of a gene between BRD and non-BRD groups, a likelihood ratio test was performed, and those genes with Benjamini-Hochberg false discovery rate (FDR) < 0.01, log fold change (log_2_FC) > 2, and log counts per million (logCPM) > 2 were identified as significant differentially expressed genes between BRD and non-BRD animals. The non-BRD/healthy control animals were set as the reference group in the contrast analysis, therefore, DE genes that were upregulated or downregulated in BRD animals were relative to the non-BRD animals.

Functional enrichment for the DE genes was performed using the Ingenuity Pathway Analysis software (IPA; www.Ingenuity.com) using their Ensembl gene ID and their log_2_ fold change as the inputs. In this study, biological functions were considered significantly enriched if the *p*-value for the overlap comparison test between the input gene list and the knowledge base of IPA for a given biological function was less than 0.05.

### eQTL analysis and eQTL annotation

We further performed eQTL analysis to identify associations between expression of differentially expressed genes and SNP genotypes. Log transformed normalized counts (log_2_CPM) values of 93 protein-coding DE genes on autosomes and 207,038 SNPs were used in the eQTL analysis. The analysis of the linear model was fitted to test the association of each single gene expression and genotype classes of a SNP implemented in the R package MatrixEQTL (Shabalin, 2012). “Feedlot”, “sequencing batch” and “genomic breed composition of animals” were also fitted in the model to correct for any variability in gene expression that could have been due to these factors. SNPs located within 1 Mbp around the gene transcription starting site (TSS) were tested for cis-associations, while SNPs located further than 1 Mbp or on other chromosomes were tested for trans-associations. Only those associations with FDR <0.05 were considered significant cis- or trans-eQTLs. The significant eQTLs were then annotated as located in the TSS-promoter, exonic, intronic, transcription termination site (TTS) or intergenic regions using the annotatePeaks.pl script of HOMER software (http://homer.ucsd.edu/homer/ngs/annotation.html).

### Sample preparation and nuclear magnetic resonance spectroscopy

The plasma metabolome was generated at The Metabolomics Innovation Centre (TMIC, Edmonton, AB, Canada). Samples underwent a deproteinization step involving ultra-filtration as previously described by Psychogios et al. (2011) to remove proteins whose presence affects the identification of small molecular weight metabolites by NMR spectroscopy. Prior to filtration, 3 KDa cut-off centrifugal filter units (Amicon Microcon YM-3) were rinsed five times each with 0.5 ml of H_2_O and centrifuged (10,000 rpm for 10 min) to remove residual glycerol bound to the filter membranes. Aliquots of each plasma sample were then transferred into the centrifuge filter devices and spun (10,000 rpm for 20 min) to remove macromolecules (primarily protein and lipoproteins) from the sample. The filtrates were checked visually for any evidence that the membrane was compromised and for such samples the filtration process was repeated with a different filter and the filtrate inspected again. The subsequent filtrates were collected, and the volumes were recorded. If the total volume of the sample was under 250 µL an appropriate amount of 150 mM KH_2_PO_4_ buffer (pH 7) was added until the total volume of the sample was 173.5 µL. Any sample that had to have buffer added to bring the solution volume to 173.5 uL was annotated with the dilution factor and metabolite concentrations were corrected in the subsequent analysis. Subsequently, 46.5 µL of a standard buffer solution (54% D_2_O:46% 1.75 mM KH_2_PO_4_ pH 7.0 v/v containing 5.84 mM DSS (2,2-dimethyl-2-silcepentane-5-sulphonate)) was added to the sample.

The sample (250 µL) was then transferred to a 3 mm SampleJet NMR tube for subsequent spectral analysis. All ^1^H-NMR spectra were collected on a 700 MHz Avance III (Bruker) spectrometer equipped with a 5 mm HCN Z-gradient pulsed-field gradient (PFG) cryoprobe. ^1^H-NMR spectra were acquired at 25°C using the first transient of the nuclear Overhauser enhancement spectroscopy (NOESY) pre-saturation pulse sequence (noesy1dpr), chosen for its high degree of quantitative accuracy (Saude et al., 2006). All FID’s (free induction decays) were zero-filled to 250 K data points. The singlet produced by the DSS methyl groups was used as an internal standard for chemical shift referencing (set to 0 ppm) and for quantification all ^1^H-NMR spectra were processed and analyzed using the Chenomx NMR Suite Professional Software package version 7.1 (Chenomx Inc., Edmonton, AB, Canada). The Chenomx NMR Suite software allows for qualitative and quantitative analysis of an NMR spectrum by manually fitting spectral signatures from an internal database to the spectrum. Specifically, the spectral fitting for metabolite was done using the standard Chenomx 700 MHz metabolite library. Typically, 90% of visible peaks were assigned to a compound and more than 90% of the spectral area could be routinely fit using the Chenomx spectral analysis software. Most of the visible peaks are annotated with a compound name. Each spectrum was processed and analyzed by at least two NMR spectroscopists to minimize compound misidentification and misquantification.

### Identification of significant metabolites and their correlations with DE genes

Fifty-four metabolites were quantified by NMR analysis followed by a logarithmic transformation to the raw data. The transformed data followed a normal distribution and were further adjusted by two fixed effects (feedlot and sex), and a covariate of genomic breed composition in linear models. The adjusted values were used to identify metabolites that had concentrations significantly (FDR <0.05) different between BRD and non-BRD animals using two independent sample t-tests. Furthermore, the correlations between 31 significant metabolites and 93 protein-coding DE genes were computed in R.

## Results

### SNPs associated with BRD susceptibility

In this study, no significant SNPs remained after FDR correction. However, two SNPs (Chr5:25858264 on chromosome 5 and BovineHD1800016801 on chromosome 18) were above the suggestive line (*p*-value <1 × 10^−5^) (Table 1; Supplementary Figure S2). Both SNPs are in intronic regions of genes, i.e., SNP Chr5:25858264 is located in the first intron of *SMUG1* while BovineHD1800016801 is located in the third intron of *IGLON5*.

**TABLE 1:** SNPs significantly associated with BRD susceptibility.

SNP	Chromosome	Position (bp)	Minor allele frequency	b	se	*p*-value
Chr5:25858264	5	25858264	0.066	1.181	0.261	5.85 × 10^−6^
BovineHD1800016801	18	57400705	0.203	0.674	0.151	7.65 × 10^−6^

### Transcriptomic architecture of BRD infection in feedlots

**FIGURE 1:**
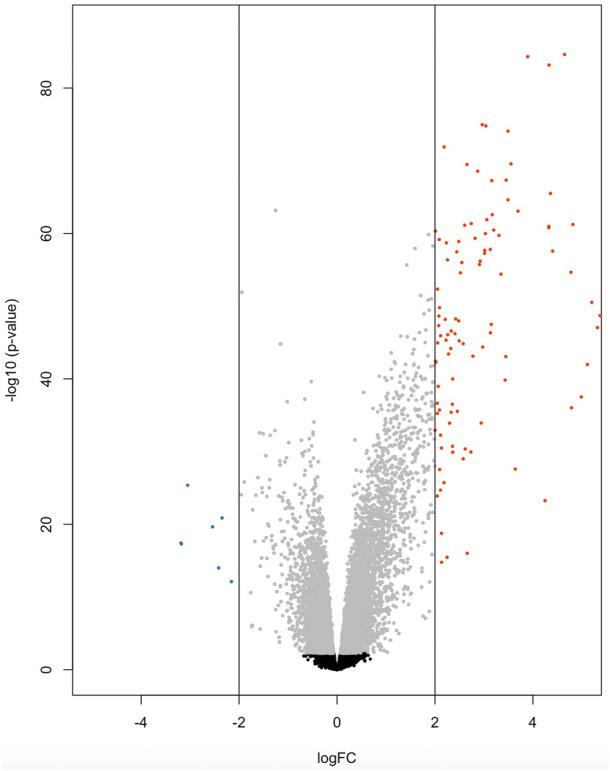
Volcano plot of 7 down-regulated genes (blue) and 94 up-regulated genes (red) for BRD animals.

At the significance threshold of log_2_FC>2, logCPM >2 and FDR <0.01, 101 genes were identified as differentially expressed between BRD and non-BRD animals (non-BRD as the reference group), of which 7 and 94 were downregulated and upregulated respectively in the infected animals (Figure 1). The full list of DE genes with related descriptions and statistics is provided in Table 2. Our result showed that *interleukin 3 receptor subunit alpha* (*IL3RA*) was the most significant (FDR = 6.6 × 10^−81^) upregulated gene, whereas *hemoglobin subunit beta* (*HBB*) was the most significant (FDR = 1.25 × 10^−24^) downregulated gene (Table 2). In terms of expression differences, *leucine rich alpha-2-glycoprotein 1* (*LRG1*) showed the highest expression (log_2_FC = 7.35) in the BRD animals relative to non-BRD animals, while *hemoglobin subunit alpha 1* (*HBA1*) showed the lowest expression (log_2_FC = -3.19) in the same animal contrast (Table 2).

**TABLE 2:** Differentially expressed genes between BRD and non-BRD animals.

Gene ID	Gene name	Log2 (fold change)	Log counts per million	*p*-value	FDR
ENSBTAG00000054844	*HBA1*	−3.188	11.000	3.80 × 10^−18^	4.95 × 10^−17^
ENSBTAG00000051412	*HBA*	−3.178	10.999	5.02 × 10^−18^	6.47 × 10^−17^
ENSBTAG00000037644	*HBB*	−3.050	11.089	4.35 × 10^−26^	1.25 × 10^−24^
ENSBTAG00000013178	*ALAS2*	−2.542	5.314	2.27 × 10^−20^	3.70 × 10^−19^
ENSBTAG00000004824	*REEP1*	−2.419	2.113	1.02 × 10^−14^	9.21 × 10^−14^
ENSBTAG00000011990	*ALOX15*	−2.347	4.915	1.35 × 10^−21^	2.46 × 10^−20^
ENSBTAG00000012403	*ARG1*	−2.157	2.589	7.74 × 10^−13^	5.65 × 10^−12^
ENSBTAG00000011465	*MYBPH*	2.002	6.087	1.16 × 10^−33^	6.80 × 10^−32^
ENSBTAG00000010464	*MN1*	2.009	2.526	4.76 × 10^−61^	3.00 × 10^−58^
ENSBTAG00000006156	*BST1*	2.012	5.490	3.76 × 10^−43^	5.60 × 10^−41^
ENSBTAG00000020430	*GLT1D1*	2.020	3.082	5.24 × 10^−43^	7.63 × 10^−41^
ENSBTAG00000050072		2.047	2.427	1.30 × 10^−24^	3.23 × 10^−23^
ENSBTAG00000052465		2.048	6.792	2.32 × 10^−37^	1.97 × 10^−35^
ENSBTAG00000013368	*ANKRD22*	2.049	3.861	5.75 × 10^−36^	4.16 × 10^−34^
ENSBTAG00000002148	*RAB3D*	2.053	7.196	4.63 × 10^−53^	1.36 × 10^−50^
ENSBTAG00000023648	*ART5*	2.055	7.114	1.13 × 10^−45^	2.00 × 10^−43^
ENSBTAG00000011037	*RBPMS2*	2.067	2.973	1.10 × 10^−39^	1.16 × 10^−37^
ENSBTAG00000039556	*WIPI1*	2.076	4.455	4.77 × 10^−48^	1.05 × 10^−45^
ENSBTAG00000006921	*ABCA6*	2.081	6.472	2.33 × 10^−49^	5.61 × 10^−47^
ENSBTAG00000013555	*ACVR1B*	2.088	5.897	6.85 × 10^−60^	3.49 × 10^−57^
ENSBTAG00000000783	*TGFA*	2.093	2.667	1.94 × 10^−36^	1.47 × 10^−34^
ENSBTAG00000004150	*NRG1*	2.093	4.792	1.59 × 10^−50^	4.13 × 10^−48^
ENSBTAG00000022779	*OLFM4*	2.097	3.277	2.97 × 10^−28^	1.04 × 10^−26^
ENSBTAG00000006990	*MYRF*	2.114	2.578	2.04 × 10^−25^	5.47 × 10^−24^
ENSBTAG00000037826		2.115	2.446	5.59 × 10^−33^	3.01 × 10^−31^
ENSBTAG00000004716	*RETN*	2.133	5.036	1.76 × 10^−19^	2.63 × 10^−18^
ENSBTAG00000014046	*BPI*	2.135	5.648	1.67 × 10^−15^	1.64 × 10^−14^
ENSBTAG00000018016	*NUPR1*	2.184	5.146	1.94 × 10^−26^	5.74 × 10^−25^
ENSBTAG00000014122	*FOXRED1*	2.188	6.744	1.28 × 10^−72^	3.39 × 10^−69^
ENSBTAG00000054765	*PGLYRP4*	2.210	2.848	6.61 × 10^−49^	1.54 × 10^−46^
ENSBTAG00000011677	*H1-2*	2.226	6.778	4.82 × 10^−46^	8.87 × 10^−44^
ENSBTAG00000013290	*DYSF*	2.233	7.225	1.98 × 10^−59^	9.37 × 10^−57^
ENSBTAG00000002635	*PGLYRP1*	2.247	2.172	3.56 × 10^−16^	3.76 × 10^−15^
ENSBTAG00000018223	*CHI3L1*	2.257	8.313	4.32 × 10^−57^	1.59 × 10^−54^
ENSBTAG00000010065	*TRPC5*	2.278	3.910	3.87 × 10^−44^	6.10 × 10^−42^
ENSBTAG00000007169	*P2RX1*	2.298	4.356	1.25 × 10^−34^	7.89 × 10^−33^
ENSBTAG00000001051	*OSCAR*	2.324	7.220	6.67 × 10^−45^	1.12 × 10^−42^
ENSBTAG00000013205	*IL1RAP*	2.331	5.612	2.63 × 10^−47^	5.44 × 10^−45^
ENSBTAG00000006904	*TNS2*	2.332	2.086	4.01 × 10^−36^	2.92 × 10^−34^
ENSBTAG00000004741	*IL12B*	2.355	2.380	3.14 × 10^−37^	2.63 × 10^−35^
ENSBTAG00000008389	*HTRA1*	2.358	2.118	1.89 × 10^−31^	8.88 × 10^−30^
ENSBTAG00000001785	*TGM3*	2.362	9.942	1.06 × 10^−40^	1.23 × 10^−38^
ENSBTAG00000018446	*GCA*	2.363	3.848	1.22 × 10^−30^	5.35 × 10^−29^
ENSBTAG00000013201	*ALOX5AP*	2.407	6.937	6.31 × 10^−47^	1.23 × 10^−44^
ENSBTAG00000020257	*PTPN5*	2.423	4.813	5.81 × 10^−49^	1.37 × 10^−46^
ENSBTAG00000018134	*AREG*	2.446	2.160	3.37 × 10^−58^	1.31 × 10^−55^
ENSBTAG00000003920	*TGM1*	2.457	2.683	2.95 × 10^−36^	2.17 × 10^−34^
ENSBTAG00000010007	*MAPK13*	2.484	4.014	1.08 × 10^−48^	2.47 × 10^−46^
ENSBTAG00000003519	*NOL3*	2.487	2.563	1.27 × 10^−59^	6.23 × 10^−57^
ENSBTAG00000005668	*SLC39A8*	2.494	2.926	5.96 × 10^−46^	1.08 × 10^−43^
ENSBTAG00000012638	*S100A12*	2.520	11.601	2.66 × 10^−55^	8.20 × 10^−53^
ENSBTAG00000008428	*UPP1*	2.549	7.233	9.94 × 10^−57^	3.47 × 10^−54^
ENSBTAG00000003353	*SLC6A2*	2.576	2.389	1.48 × 10^−45^	2.58 × 10^−43^
ENSBTAG00000048737	*DEFB10*	2.576	3.856	1.00 × 10^−29^	4.06 × 10^−28^
ENSBTAG00000006523	*SOD2*	2.607	9.705	7.47 × 10^−62^	5.21 × 10^−59^
ENSBTAG00000016566	*ITGA9*	2.618	3.070	4.37 × 10^−31^	1.98 × 10^−29^
ENSBTAG00000049416	*RAB20*	2.654	5.400	3.32 × 10^−70^	7.33 × 10^−67^
ENSBTAG00000001292	*LTF*	2.658	3.642	9.82 × 10^−17^	1.10 × 10^−15^
ENSBTAG00000021887	*DPYS*	2.734	2.126	1.13 × 10^−30^	4.97 × 10^−29^
ENSBTAG00000019669	*CD163*	2.741	8.221	4.50 × 10^−62^	3.51 × 10^−59^
ENSBTAG00000046152	*MGAM*	2.775	5.328	7.60 × 10^−44^	1.16 × 10^−41^
ENSBTAG00000013706	*MEGF9*	2.820	5.633	4.75 × 10^−60^	2.52 × 10^−57^
ENSBTAG00000017969	*CA4*	2.873	3.695	2.72 × 10^−69^	5.15 × 10^−66^
ENSBTAG00000015592	*GPR84*	2.910	3.862	1.86 × 10^−56^	6.32 × 10^−54^
ENSBTAG00000017251	*SLC26A8*	2.925	2.737	6.38 × 10^−57^	2.28 × 10^−54^
ENSBTAG00000020406	*GPC3*	2.945	3.509	1.20 × 10^−34^	7.61 × 10^−33^
ENSBTAG00000018280	*SLC28A3*	2.975	6.619	4.28 × 10^−45^	7.27 × 10^−43^
ENSBTAG00000012640	*S100A8*	3.012	10.038	5.43 × 10^−58^	2.06 × 10^−55^
ENSBTAG00000020580	*TCN1*	3.017	8.629	2.11 × 10^−58^	8.74 × 10^−56^
ENSBTAG00000006505	*S100A9*	3.030	11.263	1.04 × 10^−60^	6.26 × 10^−58^
ENSBTAG00000031950	*RAB3IP*	3.040	6.690	1.64 × 10^−75^	1.09 × 10^−71^
ENSBTAG00000019330	*PROK2*	3.058	4.710	1.28 × 10^−62^	1.06 × 10^−59^
ENSBTAG00000002233	*CPNE2*	3.130	4.908	1.61 × 10^−58^	6.88 × 10^−56^
ENSBTAG00000021240	*DCSTAMP*	3.132	4.527	4.67 × 10^−47^	9.23 × 10^−45^
ENSBTAG00000006354	*HP*	3.149	9.301	3.26 × 10^−48^	7.32 × 10^−46^
ENSBTAG00000006221	*ADGRG3*	3.160	5.550	5.38 × 10^−68^	7.92 × 10^−65^
ENSBTAG00000006999	*RYR1*	3.309	5.942	1.85 × 10^−60^	1.02 × 10^−57^
ENSBTAG00000007239	*TNFAIP6*	3.348	3.882	3.97 × 10^−55^	1.20 × 10^−52^
ENSBTAG00000020676	*MMP9*	3.434	5.948	1.48 × 10^−40^	1.66 × 10^−38^
ENSBTAG00000014149	*LCN2*	3.446	6.952	8.55 × 10^−44^	1.29 × 10^−41^
ENSBTAG00000007901	*ADGRE1*	3.454	8.940	4.73 × 10^−68^	7.83 × 10^−65^
ENSBTAG00000000377	*BMX*	3.491	5.409	8.54 × 10^−75^	3.77 × 10^−71^
ENSBTAG00000002996	*SHROOM4*	3.491	3.225	2.38 × 10^−65^	2.63 × 10^−62^
ENSBTAG00000053557	*DEFB4A*	3.640	3.012	2.60 × 10^−28^	9.14 × 10^−27^
ENSBTAG00000009773	*KREMEN1*	3.696	6.824	8.66 × 10^−64^	8.20 × 10^−61^
ENSBTAG00000049808	*IL3RA*	3.894	6.830	4.98 × 10^−85^	6.60 × 10^−81^
ENSBTAG00000048720		4.248	2.863	5.64 × 10^−24^	1.32 × 10^−22^
ENSBTAG00000008951	*ALPL*	4.327	7.425	1.10 × 10^−61^	7.29 × 10^−59^
ENSBTAG00000046158	*CFB*	4.360	5.400	3.19 × 10^−66^	3.84 × 10^−63^
ENSBTAG00000050618		4.403	4.647	2.75 × 10^−58^	1.10 × 10^−55^
ENSBTAG00000019627	*THY1*	4.777	3.655	2.17 × 10^−55^	6.85 × 10^−53^
ENSBTAG00000052012		4.788	5.793	9.54 × 10^−37^	7.57 × 10^−35^
ENSBTAG00000010273	*EREG*	4.817	2.213	5.84 × 10^−62^	4.30 × 10^−59^
ENSBTAG00000054882		4.990	4.305	3.10 × 10^−38^	2.85 × 10^−36^
ENSBTAG00000051132		5.114	5.241	1.08 × 10^−42^	1.51 × 10^−40^
ENSBTAG00000039037	*SERPINB4*	5.203	6.364	3.02 × 10^−51^	8.00 × 10^−49^
ENSBTAG00000048835		5.317	7.656	8.80 × 10^−48^	1.91 × 10^−45^
ENSBTAG00000049569		5.372	6.973	1.99 × 10^−49^	4.88 × 10^−47^
ENSBTAG00000006343	*IL1R2*	5.595	7.513	4.97 × 10^−56^	1.61 × 10^−53^
ENSBTAG00000013356	*CATHL3*	6.087	4.373	5.49 × 10^−33^	2.97 × 10^−31^
ENSBTAG00000031647	*LRG1*	7.348	4.231	2.37 × 10^−74^	7.85 × 10^−71^

**FIGURE 2:**
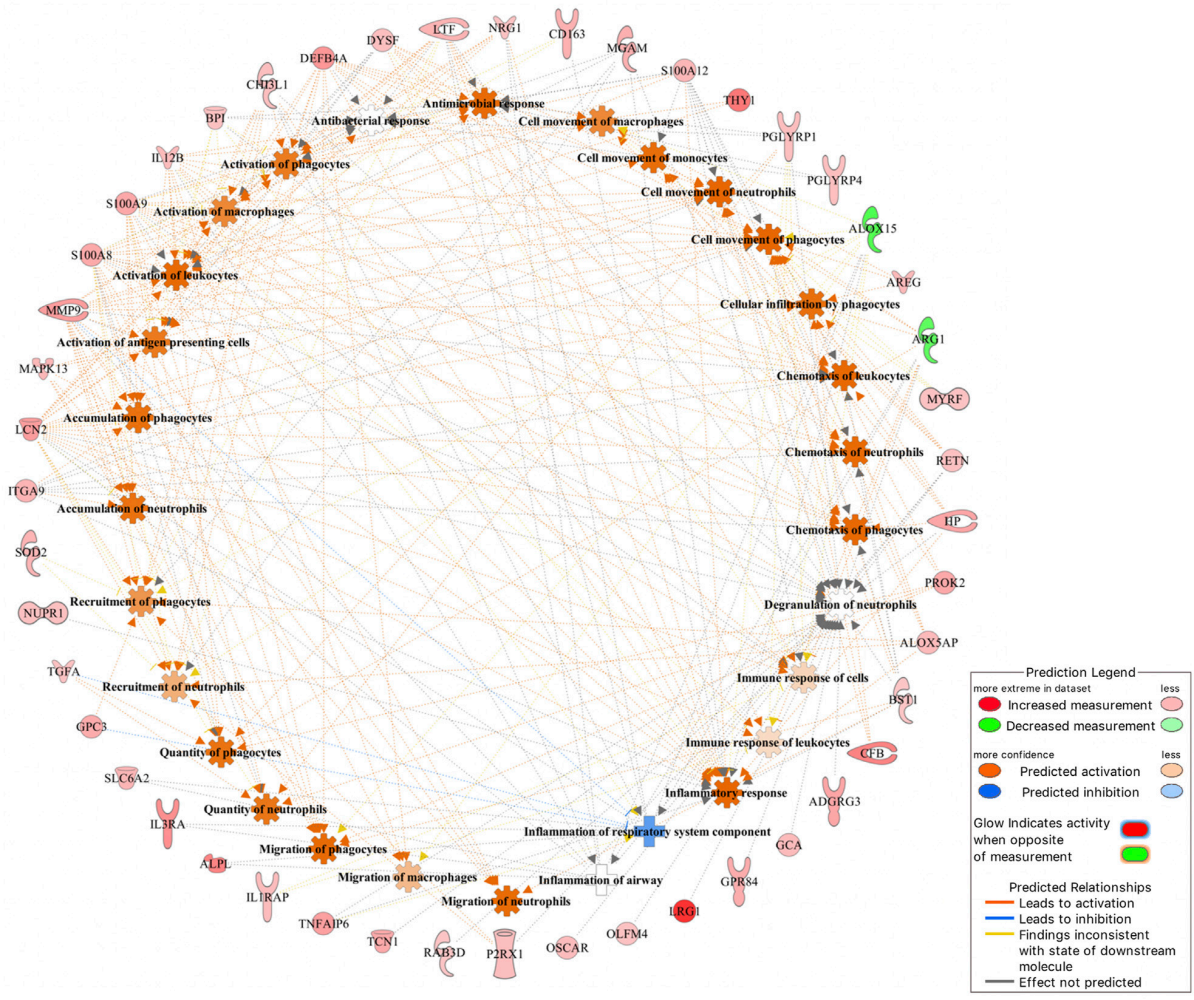
The inflammatory response was identified as the most significant (*p*-value <0.05) immune-related function that DE genes were involved in.

Of the 101 DE genes, 88 were successfully mapped to the IPA database for functional enrichment analysis. The DE genes were significantly (*p*-value <0.05) involved in 17 immune response related biological functions of which inflammatory response was the most significant with 60 DE genes. The top 10 most enriched functions are presented in Table 3, while all 17 functions are presented in Supplementary Table S1. Within the inflammatory response function, 3 DE genes (*ARG1*, *ALOX15*, and *ALAS2*) were downregulated, and 57 DE genes (e.g., *IL3RA*, *LRG1*, *BPI*, *CFB*, *GPR84*, *MMP9*, and *CA4*) were upregulated in the BRD animals. Furthermore, within the inflammatory response function, enriched innate immune response related processes such as leukocyte immune response, activation and migration of macrophages and neutrophils, and antimicrobial response were predicted to be activated or upregulated in the BRD animals (Figure 2). Adaptive immune response related processes such as activation of antigen processing cells, and cellular immune response were also identified as enriched, and predicted to be activated in the BRD animals. Some of the key DE genes as demonstrated by their involvement in numerous immune functions included *LCN2*, *S100A8*, *S100A9*, *S100A12*, *LTF*, *IL12B*, *CHI3L1*, and *DEFB4A* (Figure 2).

**TABLE 3:** Ten topmost significantly enriched biological functions associated with differentially expressed genes.

Biological function	*p*-value range	Genes involved in the biological function
Inflammatory Response	3.22 × 10^−20^–2.06 × 10^−3^	*ADGRE1, ADGRG3, ALAS2, ALOX15, ALOX5AP, ALPL, AREG, ARG1, BMX, BPI, BST1, CA4, CD163, CFB, CHI3L1, DEFB4A/DEFB4B, DPYS, DYSF, EREG, GCA, GPC3, GPR84, HP, HTRA1, IL12B, IL1R2, IL1RAP, IL3RA, ITGA9, LCN2, LRG1, LTF, MAPK13, MGAM, MMP9, MYRF, NRG1, NUPR1, OLFM4, OSCAR, P2RX1, PGLYRP1, PGLYRP4, PROK2, RAB3D, RETN, S100A12, S100A8, S100A9, SERPINB4, SLC39A8, SLC6A2, SOD2, TCN1, TGFA, TGM3, THY1, TNFAIP6, TRPC5, UPP1*
Connective Tissue Disorders	1.55 × 10^−14^–1.62 × 10^−3^	*ALAS2, ALOX15, ALOX5AP, ALPL, AREG, ARG1, BMX, BPI, CA4, CD163, CFB, CHI3L1, DCSTAMP, DPYS, GCA, GPC3, HP, HTRA1, IL12B, IL1R2, IL3RA, ITGA9, KREMEN1, LCN2, LTF, MMP9, PGLYRP1, PROK2, RETN, S100A12, S100A8, S100A9, SLC39A8, SLC6A2, SOD2, TGFA, TNFAIP6*
Inflammatory Disease	1.55 × 10^−14^–1.83 × 10^−3^	*ADGRE1, ALAS2, ALOX15, ALOX5AP, ALPL, AREG, ARG1, BMX, BPI, CA4, CD163, CFB, CHI3L1, DEFB4A/DEFB4B, DPYS, EREG, GCA, H1-2, HP, HTRA1, IL12B, IL1R2, IL3RA, ITGA9, LCN2, LRG1, LTF, MGAM, MMP9, NRG1, OLFM4, PGLYRP1, PGLYRP4, PROK2, RETN, S100A12, S100A8, S100A9, SERPINB4, SLC39A8, SLC6A2, SOD2, TCN1, TGFA, TGM3, THY1, TNFAIP6*
Organismal Injury and Abnormalities	1.55 × 10^−14^–2.07 × 10^−3^	*ABCA6, ACVR1B, ADGRE1, ADGRG3, ALAS2, ALOX15, ALOX5AP, ALPL, ANKRD22, AREG, ARG1, ART5, BMX, BPI, BST1, CA4, CD163, CFB, CHI3L1, CPNE2, DCSTAMP, DEFB4A/DEFB4B, DPYS, DYSF, EREG, FOXRED1, GCA, GLT1D1, GPC3, GPR84, H1-2, HBD, HP, HTRA1, IL12B, IL1R2, IL1RAP, IL3RA, ITGA9, KREMEN1, LCN2, LRG1, LTF, MAPK13, MEGF9, MGAM, MMP9, MN1, MYBPH, MYRF, NOL3, NRG1, NUPR1, OLFM4, OSCAR, P2RX1, PGLYRP1, PGLYRP4, PROK2, PTPN5, RAB20, RAB3D, RAB3IP, RBPMS2, REEP1, RETN, RYR1, S100A12, S100A8, S100A9, SERPINB4, SHROOM4, SLC26A8, SLC28A3, SLC39A8, SLC6A2, SOD2, TCN1, TGFA, TGM1, TGM3, THY1, TNFAIP6, TNS2, TRPC5, UPP1, WIPI1*
Immunological Disease	3.58 × 10^−11^–2.05 × 10^−3^	*ADGRG3, ALAS2, ALOX15, ALOX5AP, ALPL, AREG, ARG1, BMX, BPI, CD163, CFB, CHI3L1, DEFB4A/DEFB4B, GCA, GPC3, GPR84, HP, IL12B, IL1R2, IL3RA, ITGA9, LCN2, LTF, MGAM, MMP9, NRG1, PGLYRP1, PROK2, RETN, S100A12, S100A8, S100A9, SERPINB4, SLC6A2, SOD2, TGFA, TGM3, TNFAIP6*
Infectious Diseases	1.62 × 10^−8^–1.42 × 10^−3^	*ALOX5AP, ALPL, BPI, CD163, CFB, DEFB4A/DEFB4B, DYSF, GCA, GPC3, H1-2, HP, IL12B, IL1R2, IL3RA, LCN2, LTF, MGAM, MMP9, MYRF, NRG1, OLFM4, P2RX1, PGLYRP1, RAB3D, RETN, S100A12, S100A8, S100A9, SLC6A2, TCN1*
Respiratory Disease	1.62 × 10^−8^–1.73 × 10^−3^	*ABCA6, ACVR1B, ALAS2, ALOX15, ALPL, ANKRD22, AREG, ARG1, BMX, BPI, BST1, CA4, CD163, CFB, CHI3L1, CPNE2, DCSTAMP, DPYS, DYSF, EREG, FOXRED1, GLT1D1, GPC3, GPR84, H1-2, HP, HTRA1, IL12B, IL1RAP, IL3RA, ITGA9, LCN2, LTF, MAPK13, MEGF9, MGAM, MMP9, MN1, MYBPH, MYRF, NOL3, NRG1, NUPR1, OLFM4, PGLYRP1, PGLYRP4, PTPN5, RETN, RYR1, S100A12, S100A8, S100A9, SERPINB4, SHROOM4, SLC6A2, SOD2, TCN1, TGFA, TGM3, THY1, TNFAIP6, TNS2, TRPC5*
Antimicrobial Response	2.01 × 10^−8^–2.57 × 10^−4^	*BPI, DEFB4A/DEFB4B, IL12B, LCN2, LTF, PGLYRP1, PGLYRP4, S100A12, S100A8, S100A9*
Psychological Disorders	2.55 × 10^−8^–1.83 × 10^−3^	*ALOX15, ARG1, CA4, CFB, CHI3L1, DYSF, HP, HTRA1, IL12B, IL1R2, LCN2, LRG1, LTF, MMP9, NRG1, PTPN5, RYR1, S100A9, SLC6A2, SOD2, TGM1, THY1, UPP1*
Metabolic Disease	1.81 × 10^−7^–7.5 × 10^−4^	*ALOX15, ALOX5AP, ALPL, ARG1, BPI, CA4, CFB, CHI3L1, DYSF, GPC3, HBD, HP, HTRA1, IL12B, IL1R2, IL3RA, LCN2, LTF, MGAM, MMP9, PTPN5, RETN, S100A8, S100A9, SLC6A2, SOD2, TGM1, THY1*

### Gene expression and genotype associations

**FIGURE 3:**
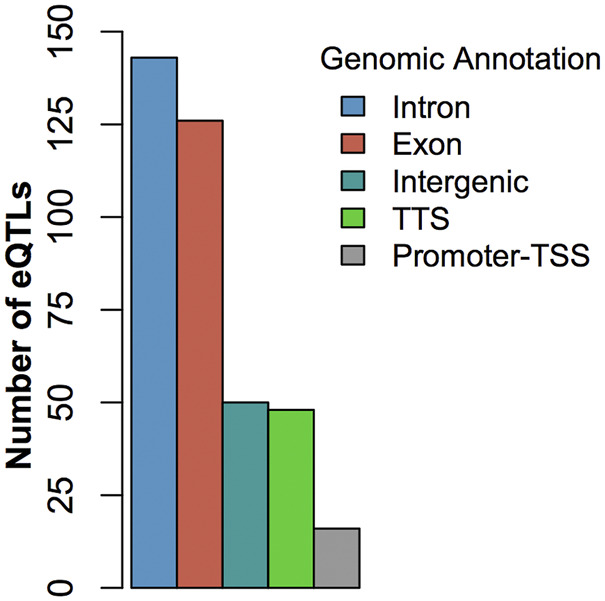
Histogram of the distribution of eQTLs by genic regions.

At FDR <0.05, we identified 420 cis-eQTLs and 144 trans-eQTLs associated with the expression of DE genes (Supplementary Table S2, S3). Some cis-eQTLs and trans-eQTLs were associated with more than one DE gene associated with BRD. For example, the SNP Chr6:110850346 was cis-eQTL associated with the expression of the DE gene *BST1* and a trans-eQTL associated with the expression of another 6 DE genes (*GPR84*, *NUPR1*, *ART5*, *CFB*, *SLC6A2*, and *ADGRE1*). Similarly, the expression of a DE gene could also be associated with more than one cis- or trans-eQTLs. Of note, the eQTL analysis showed that the SNP (Chr5:25858264) with the smallest *p*-value in GWAS (Table 1) was cis-eQTL associated with the expression of the DE gene *GPR84* (Supplementary Table S2, S3). Additionally, 2 potential trans-eQTL hotspots (rs207554348 on chromosome 3 and Chr2:118164919 on chromosome 2) were observed (Supplementary Table S3). Finally, the eQTL annotation showed that the eQTL SNPs identified in this study were mostly located in intronic and exonic regions rather than intergenic regions (Figure 3; Supplementary Table S4). The high proportion of eQTLs observed in intronic and exonic regions may be due to the uneven distribution of SNPs used in this study, with 58.8% of SNPs derived from RNA-seq data.

### Metabolites associated with BRD infection and correlations between metabolites and DE genes

A total of 31 metabolites showed significant abundance difference between BRD and non-BRD animals (Table 4). Twenty metabolites had lower abundance in BRD animals as compared to non-BRD animals, for example, citric acid showed the most significant difference, and was significantly more abundant in non-BRD animals (Table 4). However, we also observed a few metabolites that were more strongly abundant in BRD animals than non-BRD animals, such as d-mannose, l-phenylalanine, and l-carnitine. In addition, 17 significant metabolites also showed significant (FDR <0.05) correlations with the expression of DE genes (Supplementary Table S5). It is worth noting that 15 metabolites had significant correlations with over 74 DE genes, with citric acid, 3-hydroxybutyric acid, acetic acid, l-glutamic acid, d-mannose, l-carnitine, showing correlation with more than 90 DE genes. For those metabolites that were more significantly associated with BRD, they also have a greater number of correlations with DE genes. With respect to DE genes, our results also showed that most (88) of the DE genes had significant correlations with more than 10 metabolites associated with BRD. Seventy percent of significant correlations between DE genes and metabolites were negative. In other words, in general, BRD animals had higher expression of DE genes and lower concentrations of metabolites in blood tissues. However, there are some interesting exceptions, such as d-mannose, l-phenylalanine, and l-carnitine, which were positively correlated with DE genes and also more abundant in BRD animals.

**TABLE 4:** Significant metabolites between BRD and non-BRD animals.

Metabolite	t-value	*p*-value	FDR
Citric acid	−20.226	5.71 × 10^−39^	3.08 × 10^−37^
d-Mannose	12.979	1.28 × 10^−21^	3.45 × 10^−20^
Acetic acid	−12.277	7.88 × 10^−20^	1.42 × 10^−18^
Isobutyric acid	−11.291	2.62 × 10^−17^	3.54 × 10^−16^
l-Phenylalanine	97.038	2.89 × 10^−14^	3.12 × 10^−12^
3-Hydroxybutyric acid	−94.871	1.01 × 10^−12^	9.12 × 10^−12^
l-Glutamine	−89.717	1.97 × 10^−11^	1.52 × 10^−10^
l-Glutamic acid	−86.547	1.20 × 10^−10^	8.08 × 10^−10^
Tyrosine	−7.910	7.71 × 10^−10^	4.63 × 10^−8^
l-Carnitine	78.243	1.24 × 10^−9^	6.67 × 10^−8^
Betaine	−71.983	3.65 × 10^−7^	1.79 × 10^−6^
l-Threonine	−62.151	5.78 × 10^−5^	2.60 × 10^−4^
Isopropanol	38.856	1.58 × 10^−4^	4.27 × 10^−4^
Malonate	−36.215	4.11 × 10^−4^	1.06 × 10^−3^
Glycine	−35.156	5.95 × 10^−4^	1.46 × 10^−3^
l-Acetylcarnitine	57.995	4.39 × 10^−4^	1.82 × 10^−3^
l-Serine	−32.186	1.61 × 10^−3^	3.78 × 10^−3^
Methionine	−2.899	4.36 × 10^−3^	9.81 × 10^−3^
l-Proline	−5.404	2.81 × 10^−3^	1.08 × 10^−2^
Dimethylglycine	27.494	6.78 × 10^−3^	1.42 × 10^−2^
Choline	−27.472	6.82 × 10^−3^	1.42 × 10^−2^
2-Hydroxyisovalerate	27.148	7.48 × 10^−3^	1.50 × 10^−2^
l-Aspartate	−26.911	8.01 × 10^−3^	1.54 × 10^−2^
l-Lactic acid	25.391	1.22 × 10^−2^	2.28 × 10^−2^
Valine	−24.949	1.38 × 10^−2^	2.48 × 10^−2^
l-Leucine	24.787	1.44 × 10^−2^	2.51 × 10^−2^
Acetone	23.818	1.86 × 10^−2^	3.14 × 10^−2^
l-Lysine	22.934	2.34 × 10^−2^	3.82 × 10^−2^
Urea	−22.647	2.51 × 10^−2^	3.99 × 10^−2^
l-Asparagine	−50.932	1.14 × 10^−2^	4.11 × 10^−2^
Sarcosine	−21.973	2.97 × 10^−2^	4.58 × 10^−2^

## Discussion

The BRD and non-BRD animals used in the current study were fed and raised in the same feedlots under similar management and environmental factors. It is therefore expected that all animals in the study were equally exposed to BRD causing pathogens, hence, all BRD animals are assumed to be susceptible while non-BRD animals are resistant. Disease susceptibility and resistance were defined in relation to BRD in general as a multifactorial and multi-pathogen disease, and not according to specific pathogens. Based on this assumption, two SNPs (Chr5:25858264 and BovineHD1800016801) associated with BRD susceptibility in beef cattle were identified through GWAS (Table 1). The most significant SNP (Chr5:25858264) explained 17% of the phenotypic variance for BRD susceptibility. This implies that this SNP could be a major quantitative trait nucleotide or in linkage disequilibrium with a major QTL for BRD susceptibility in the studied population. However, the proportion of phenotypic variance explained by significant SNPs in the current study might have been overestimated because of the limited number of animals used. In addition, the low coverage depth of SNP calling and low minor allele frequency of Chr5:25858264 may have also led to a false-positive result. Thus, future research utilizing a larger sample size and higher whole genome sequencing depth are warranted to provide more power for fine mapping this QTL and identifying the causal gene and mutation for BRD resistance.

In addition, results from our study revealed substantial expression differences of 101 genes in the blood tissue of BRD and healthy animals. About 93% of these DE genes were upregulated in the BRD animals (Table 2). Among these upregulated genes, *IL3RA* and *LRG1* showed the strongest association with BRD in terms of statistical significance and fold change, respectively. *IL3RA* encodes the protein of interleukin 3 receptor subunit alpha which is a cytokine receptor protein for interleukin 3 (IL3), colony stimulating factor 2 (CSF2/GM-CSF) and interleukin 5 (IL5) (Milatovich et al., 1993). The cytokine IL3 is generated from T-cells and stem cells, and is involved in macrophage activation and regulation of cytokine production (Frendl, 1992). On the other hand, IL-5 is produced by CD4^+^ T-cells and causes B-cell growth factor and differentiation, IgA selection, eosinophil activation, and increased production of innate immune cells (Akdis et al., 2011). This study also identified DE genes (*IL1R2*, *IL1RAP*, and *IL12B*) related to interleukin-1 (IL-1) and interleukin-12 (IL-12) which cause lymphocyte activation, macrophage stimulation, increased leukocyte adhesion and release of acute phase proteins by the liver, or induced interferon gamma production by T-cells and natural killer cells (Arena et al., 1998; Akdis et al., 2011; Dinarello, 2018; Jiang et al., 2018). It is worth noting that *IL3RA* and *LRG1* have been reported to be associated with BRD in previous transcriptomic studies (Tizioto et al., 2015; Scott et al., 2020; Jiminez et al., 2021). *LRG1* encodes the protein of leucine rich alpha-2-glycoprotein one that has been reported to be packaged into the granule compartment of human neutrophils and secreted upon neutrophil activation (Druhan et al., 2017). For downregulated genes, the top 3 genes (*HBA1*, *HBA*, and *HBB*) are all related to hemoglobin–the oxygen-carrying protein within red blood cells. Specifically, *HBA1* and *HBA* encode for α-globin, and *HBB* encodes β-globin, which are the two main globins that compose hemoglobin (Marengo-Rowe, 2006). Thus, the low expressed level of *HBA1*, *HBA*, and *HBB* along with inflammation may indicate anemia of inflammation in infected cattle (BRD susceptible cattle). Additionally, anemia of inflammation could cause normal or sometimes increased amount of iron stored in tissues, but a low level of iron in blood (Nemeth and Ganz, 2014; Fraenkel, 2017). Iron homeostasis is involved in oxygen transport, cellular respiration, and metabolic processes (Ali et al., 2017). The regulation of iron concentration in blood also plays an important role in modulating bacterial infection and contributes to the progression of lung disease (Roehrig et al., 2007; Ali et al., 2017), which could be one of the factors associated with animal susceptibility to BRD. Future studies should determine the relationship between iron levels and susceptibility to BRD in feedlot animals to investigate this hypothesis.

Some of the DE genes identified in the current study have been identified as associated with BRD in beef cattle in other similar studies investigating the lymph node tissue (Tizioto et al., 2015) bronchial epithelial cells (N’jai et al., 2013), and blood (Scott et al., 2020; Jiminez et al., 2021). For example, compared with the results of Tizioto et al. (2015), 26, 35, 29, 39, 20, and 8 of DE genes identified in this study were common with those identified in the lymph node of animals who were challenged by BRSV, IBR, BVDV, *M. haemolytica, P. multocida, and M. bovis*, respectively (overlapping genes are shown in the Supplementary Table S6). In addition to identifying DE genes specific to individual challenges, Tizioto et al. (2015) found 25 genes expressed differentially in all the infections, of which 5 genes (*S100A8, S100A9, MMP9, TGM3, and PGLYRP1*) were also identified as DE genes in the current study. These genes may be differentially expressed in all pathogen challenges because they are related to innate immune cells. For example, *S100A8 and S100A9* are expressed in neutrophils and monocytes (Edgeworth et al., 1991) and are known danger-associated molecular patterns that activate innate immune response by binding to pattern recognition receptors of the innate immune cells in response to pathogenic attacks (Schiopu and Cotoi, 2013). Additionally. N’jai et al. (2013) reported the top 70 DE genes identified in bovine bronchial epithelial cells, and three of these genes (*CA4, TNFAIP6, and HP*) were identified in our current study, as well as previous studies (Tizioto et al., 2015; Jiminez et al., 2021). Comparing our results with DE genes identified in blood samples from other studies (Scott et al., 2020; Jiminez et al., 2021), more common DE genes, such as *LRG1, CFB, and ALOX15*, were observed. This reveals that the DE genes associated with BRD in different populations are relatively consistent. Furthermore, BRD is a polymicrobial disease that is usually the result of co-infection of several common viral and bacterial pathogens (Dabo et al., 2008; Rice et al., 2008; Griffin et al., 2010; Klima et al., 2014). The infection of different pathogens may cause different immune responses and result in related gene expression in the host (N’jai et al., 2013; Tizioto et al., 2015). When comparing the results of this study to those of Tizioto et al. (2015), the infection process in our population seems to involve multiple pathogens as well. However, the expression of some genes is associated with more than one pathogen and some genes are expressed in response to all pathogen infections (N’jai et al., 2013; Tizioto et al., 2015) makes it difficult to distinguish specific pathogen infections based on gene expression alone. In this study, the study design and the objectives were to determine common immune responses to BRD infection and to identify DE genes that could be used in different populations and feedlots. Future studies to evaluate the influence of specific pathogens on gene expression in blood are recommended. This may help identify pathogen-specific DE genes for better control and treatment of BRD.

Investigation into the biological involvement of the DE genes revealed inflammatory response as the most significant enriched function. In animals, inflammatory response is a biological response of the immune system to injurious stimuli, such as pathogen presence, damaged cells and toxic compounds (Ferrero-Miliani et al., 2007; Medzhitov, 2010). This response is aimed at clearing the immune insulting agents and initiating healing (Ferrero-Miliani et al., 2007; Medzhitov, 2010). Upon recognition of the pathogenic agents, the immune system responds to such attack by recruiting and activating the phagocytic cells such as macrophages and neutrophils, and those phagocytes that are tasked with the immediate destruction and clearing of the pathogenic agents from the body (Ackermann et al., 2010; Mantovani et al., 2011). Interestingly, activation and recruitment of both neutrophils and macrophages were among the processes identified as enriched within the inflammatory response in the current study (Figure 2). These processes were predicted to be activated in the BRD animals compared to the non-BRD animals, indicating that the inflammatory response plays a key role in the defense against BRD pathogenic infection. Previous transcriptome studies of blood and other immune organs also demonstrated the significant association of the inflammatory response with BRD status (N’jai et al., 2013; Scott et al., 2020). Some of the interesting inflammatory response genes involved in multiple innate immune responses include *LCN2, S100A8, S100A9, S100A12, LTF, IL12B, CHI3L1, DEFB4A, and MMP9*. In line with our results and our interpretation of these results, Tizioto et al. (2015) reported DE genes and pathways that were found to be common to all pathogen challenges that were upregulated (e.g., *S100A8, S100A9, and MMP9*) in the challenged (BRD) animals and appear to primarily be related to the innate immune response. Additionally, we also observed that some genes, such as *LCN2 and LTF* were predicted to be involved in the cell-mediated immune response, indicating they may be key genes to against viral infections. Therefore, functional enrichment analyses of DE genes provided insights into the biological background of BRD infection and host immune response.

**FIGURE 4:**
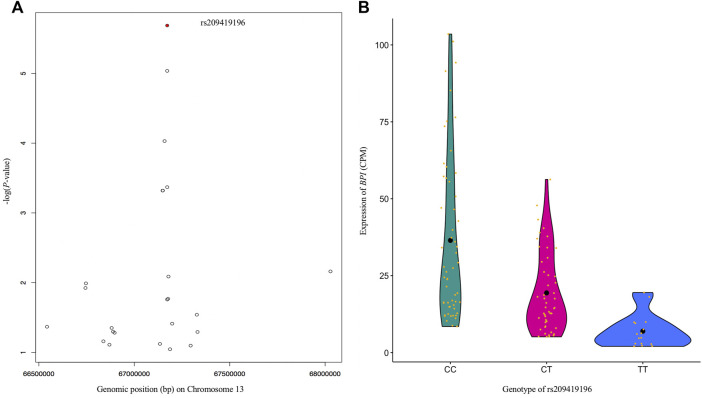
Regional Manhattan plot **(A)** for all SNPs around 1 Mbp up- and down-stream of *BPI*. SNP rs209419196 (red dot) is the most significant SNP associated with expression of *BPI*. Violin plot **(B)** of the effects of three genotypes (CC, CT, and TT) of rs209419196 on the expression of *BPI*. The differences in *BPI* expression among CC, CT, and TT were significant (*p*-value <0.05).

As BRD is caused by multiple viral and bacterial pathogens, the identification of DE genes associated with immune responses to pathogens was in line with our expectations. To study the associations between DNA markers and gene expression, and potential complex regulations of gene expression, we performed eQTL analysis. The information obtained from eQTL analysis could help to understand the GWAS results and illustrate the causality between the significant SNP and BRD susceptibility. For example, the SNP (Chr5:25858264) with the lowest *p*-value in this GWAS showed a cis-effect on the DE gene *GPR84* (Supplementary Table S2). The expression of *GPR84* was mainly observed in bone marrow, lung, and peripheral blood leukocytes (Yousefi et al., 2001), and identified in cells of both the innate and adaptive immune system, which plays a role in pro-inflammatory responses, e.g., cytokine production (Alvarez-Curto and Milligan, 2016; Zhang et al., 2016). The SNP Chr5:25858264 is located downstream of *GPR84*, we speculate the genomic region spanning the SNP might be hosting an enhancer for *GPR84* and hence modulate its expression. However, further experimental evidence is needed to support this speculation. The essential role of eQTL in explaining genetic variance and the shaping of beef cattle phenotypes has been previously reported (Xiang et al., 2022). Additionally, these results obtained from eQTL analysis could help to pinpoint causal SNPs associated with susceptibility to BRD. For example, Neibergs et al. (2014b) reported a genomic region covering *BPI* as associated with BRD susceptibility in Holstein calves, thus indicating that variants within or near this gene have functional relevance in modulating susceptibility to BRD in cattle. *BPI* was also a DE gene associated with BRD in both the current and previous studies (Tizioto et al., 2015; Jiminez et al., 2021). *BPI* encodes the bactericidal permeability increasing protein, a critical protein involved in neutralizing Gram-negative bacteria lipopolysaccharide antigen while mediating and promoting Gram-negative bacteria recognition by monocytes for phagocytosis (Yu and Song, 2020). Through the eQTL analysis, we further identified another likely causal SNP among all variants within or near the gene *BPI*. The SNP (rs209419196) was the most significant SNP (*p*-value < 2.1×10^−6^, FDR<0.006) among six cis-eQTLs associated with the expression of *BPI* (Figure 4A), and the expression of *BPI* was significantly (*p*-value <0.05) decreased as the number of “T” alleles increased in the genotype (Figure 4B). According to the eQTL annotation analysis, rs209419196 was predicted to be in the promoter region of *BPI*, which is located 92 bp downstream of the 5’ end of the transcription start site for the transcript (ENSBTAT00000077785) of *BPI*. Therefore, the results for eQTLs and their annotation not only provide important reference information for GWAS interpretation and causal SNP identification, but also provide additional insights into potential molecular mechanisms of differential gene expression related to disease state. Furthermore, the identification of these SNP markers provides more functional information that can be utilized to enhance genomic selection for BRD resistance in beef cattle.

As BRD is a complex pathogenic interaction with multiple etiologies and risk factors, it is difficult to control and prevent. Conventionally, BRD diagnosis is based on clinical signs, and varies among environment, calf caretakers, producers, and herd veterinarians, often causing a high proportion of false-negative and false-positive diagnoses (Moisá et al., 2019). Such diagnostic inaccuracies lead to the progression of disease, misuse of antimicrobials, production losses, and suboptimal animal welfare outcomes (Moisá et al., 2019). Therefore, accurate diagnostic methods for BRD are still needed. Blood transcriptomic and metabolomic biomarkers have been proposed to be used in the identification of BRD cattle in feedlots (Blakebrough-Hall et al., 2020; Sun et al., 2020). In this study, 101 DE genes were identified and the most informative marker *LRG1* has been previously identified as a potential biomarker for different infections (e.g., active tuberculosis) in humans (Wu et al., 2015; Fujimoto et al., 2020; Yang et al., 2020; Ma et al., 2021). We also found 31 metabolites were significantly associated with BRD infection, 13 of which were consistent with significant metabolites (e.g., citric acid, d-mannose, and acetic acid) identified by Blakebrough-Hall et al. (2020), even though the metabolite profiles used in the two studies were not exactly the same. In addition, 17 significant metabolites were significantly correlated with DE genes and the majority of correlations were negative (Supplementary Table S5), suggesting that generally increased expression of DE genes could result in decreased concentration/abundance of BRD-associated metabolites in blood tissues of BRD animals, thereby negatively impacting the metabolism of sick animals. Therefore, we suggest that combining such transcriptomic and metabolomic signatures may be useful for BRD identification in feedlots. However, validation in other independent beef cattle populations is required before evaluating their performance and practicality.

## Conclusion

Genomic, transcriptomic and metabolomic data were applied here to elucidate the genetic and molecular background of BRD infection in feedlot beef cattle. Two SNPs associated with BRD susceptibility were identified through GWAS. Transcriptomic and functional analyses revealed 101 DE genes associated with BRD infection. These genes were mainly involved in inflammatory response processes such as recruitment and activation of phagocytes. The most significant SNP (Chr5:25858264) from the GWAS analysis was also a cis-eQTL associated with a DE gene *GPR84*. This indicates that our integrative analyses could help with the refining of GWAS results and the identification of causal SNPs associated with BRD susceptibility. Additionally, we found 31 metabolites associated with BRD infection and 17 of them were correlated with many DE genes, which indicated the potential biological connections between DE genes and metabolites. Overall, this preliminary multi-omics study illustrates the complex relationships among different omics, which could improve the understanding of genetic and molecular mechanisms underlying the disease.

## Data Availability

The RNA data presented in the study are deposited in the NCBI Gene Expression Omnibus (GEO) database, accession number GSE217317. The genotype data, phenotype data, and metabolomic data presented in the study are deposited in the Borealis database, doi:10.5683/SP3/ZETWNY.
