# Supplementary figures and images for: Applying multi-omics data to study the genetic background of bovine respiratory disease infection in feedlot crossbred cattle
Source: Front Genet. 2022 Dec 12;13:1046192. doi: 10.3389/fgene.2022.1046192 (PMC9790935; doi:10.3389/fgene.2022.1046192)

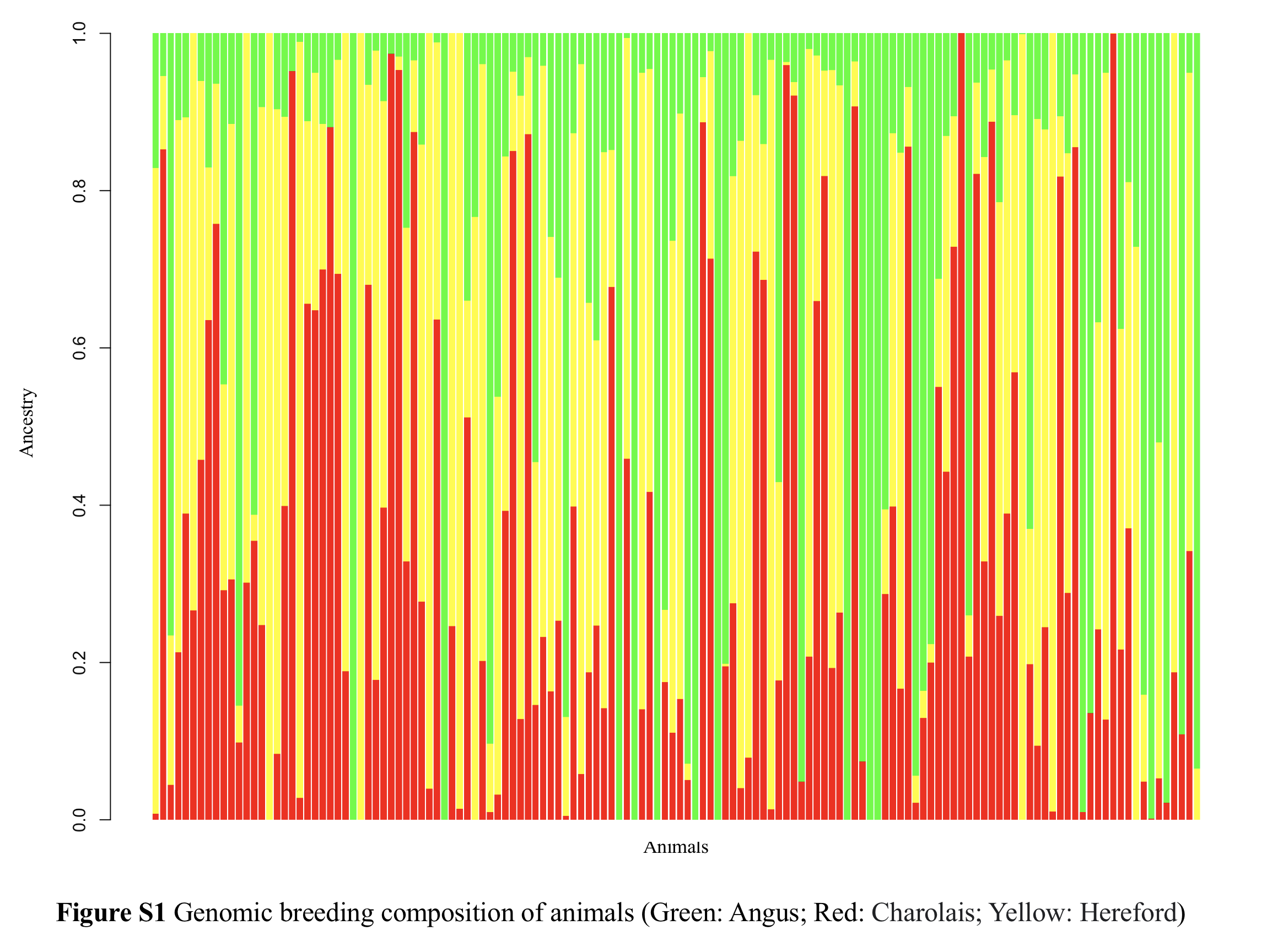

Supplement: Supplementary file 1 [file Image1.JPEG]

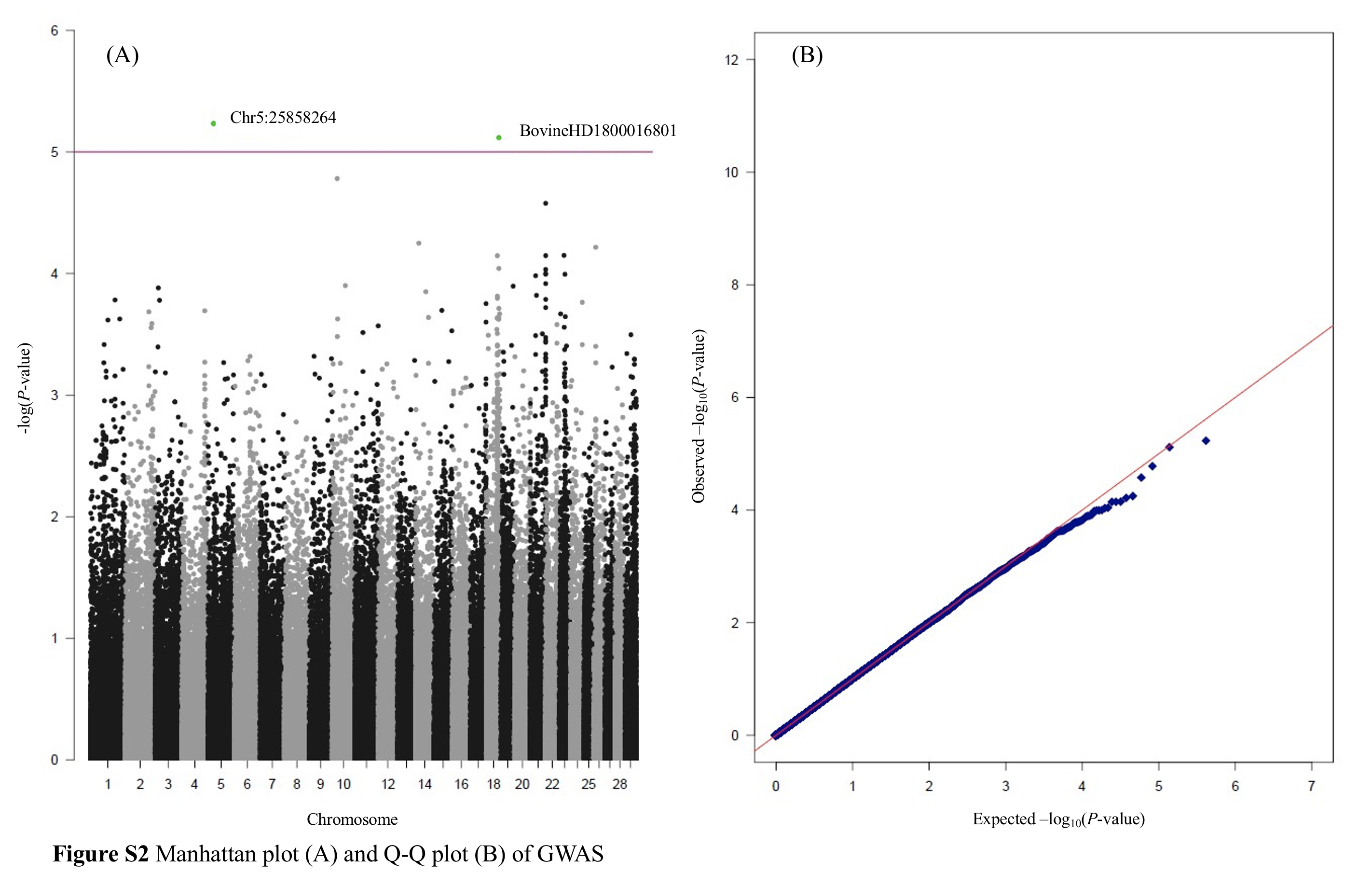

Supplement: Supplementary file 2 [file Image2.JPEG]
